# Effect of acute intravenous beta-blocker administration on myocardial blood flow during same-day hybrid CCTA/PET imaging

**DOI:** 10.1007/s10554-024-03212-w

**Published:** 2024-08-05

**Authors:** Marko Gajic, Andrei Galafton, Pascal S. Heiniger, Tobia Albertini, Stjepan Jurisic, Catherine Gebhard, Dominik C. Benz, Aju P. Pazhenkottil, Andreas A. Giannopoulos, Philipp A. Kaufmann, Ronny R. Buechel

**Affiliations:** Department of Nuclear Medicine, Cardiac Imaging, University and University Hospital Zurich, NUK A 12 Ramistrasse 100, 8091 Zurich, Switzerland

**Keywords:** Hybrid imaging, Coronary CT angiography, Positron emission tomography, Myocardial blood flow, Beta-blockers, Intravenous

## Abstract

This study aimed to evaluate the impact of acute intravenous beta-blocker administration on myocardial blood flow (MBF) during same-day hybrid coronary computed tomography angiography (CCTA) and 13N-ammonia positron emission tomography (PET) myocardial perfusion imaging (MPI). Previous research on the discontinuation of oral beta-blockers before MPI has shown mixed results, with no studies yet exploring the acute intravenous administration in the context of same-day hybrid imaging. This retrospective study included patients with suspected chronic coronary syndromes undergoing same-day hybrid CCTA/13N-ammonia PET MPI. Exclusion criteria comprised coronary artery stenosis ≥ 50% or regional perfusion abnormalities on PET, and baseline oral beta-blocker medication. Intravenous metoprolol (up to 30 mg) was administered as needed for heart rate control before CCTA. MBF measurements were obtained at rest (rMBF) and during stress (sMBF), and myocardial flow reserve (MFR) was calculated. After excluding 281 patients, 154 were eligible for propensity-score matching, resulting in 108 patients divided into two equal groups based on beta-blocker administration. The groups showed no significant differences in baseline characteristics. Among those who received beta-blockers, there was a significant decrease in sMBF (2.21 [IQR 1.72–2.78] versus 2.46 [2.08–2.99] ml∙min^−1^∙g^−1^, p = 0.027) and MFR (3.46 [2.70–4.05] versus 3.79 [3.22–4.46], p = 0.030), respectively, compared to those who did not receive beta-blockers. In contrast, rMBF remained unaffected (0.65 [0.54–0.78] versus 0.64 [0.55–0.76] ml∙min^−1^∙g^−1^, p = 0.931). Acute intravenous beta-blocker administration significantly impacts MBF, leading to a slight reduction in sMBF and MFR. In contrast, rMBF appears unaffected, suggesting that beta-blockers primarily affect the coronary capacity to respond to vasodilators.

**Supplementary Information:**

The online version contains supplementary material available at 10.1007/s10554-024-03212-w.

## Introduction

The value of cardiac hybrid imaging with coronary computed tomography angiography (CCTA) and positron emission tomography (PET) myocardial perfusion imaging (MPI) lies in its comprehensive diagnostic capability, combining precise anatomical details of coronary arteries from CCTA with functional insights from PET MPI [1]. Current guidelines for chronic coronary syndrome (CCS) recommend CCTA for initial diagnosis. For further evaluation of lesions’ hemodynamic significance, hybrid CCTA/PET myocardial perfusion imaging (MPI) has been shown to provide clinical value [2, 3]. To obtain high-quality images of coronary arteries with CCTA, controlling the patient’s heart rate (HR) to remain low and regular is crucial. In clinical practice, patients frequently present with a high resting HR or with various irregular heart rhythms, including premature contractions and different forms of tachycardia, mandating the administration of beta-blockers to lower and stabilize the HR to ensure diagnostic image quality in the vast majority of patients. [4, 5]. On the other hand, some studies have demonstrated that administration of beta-blockers may modify the results of stress myocardial perfusion imaging (MPI), potentially decreasing the sensitivity of MPI to detect ischemia [6]. In case of physical exercise, the well-known anti-ischemic effects of beta-blockers, namely a decrease of myocardial oxygen demand through a reduction in HR, blood pressure (BP), and myocardial contractility, may explain their impact on MPI findings. In case of vasodilator stress, however, the evidence remains scarce, and the results are sometimes contradictory, with the majority of these data stemming from small historical studies, focusing primarily on the impact of withdrawal from baseline oral beta-blocker medication on single photon emission computed tomography (SPECT) [6]. To the best of our knowledge, no study to date has addressed the effects of acute, i.e. same-day and intravenous application of beta-blockers on quantitative myocardial blood flow (MBF). As the adoption of PET MPI is strongly increasing not only in the United States but following key regulatory approvals in several countries, also in Europe, it becomes evident that we are in need of a better understanding of the interplay between beta-blockers and MBF measurements, particularly given that more and more centers tend to perform same-day sequential or hybrid CCTA/PET MPI. In the present study, we aimed to assess the potential impact of acute intravenous administration of beta-blockers on MBF as assessed by 13N-ammonia PET myocardial perfusion imaging.

## Methods

### Patient population

This is a retrospective single-center study. From our institutions’ electronic medical records, we identified patients who underwent same-day hybrid CCTA/13N-ammonia PET MPI for evaluation of suspected CCS at our Department between November 2013 and June 2023. We then identified and excluded patients in whom CCTA was performed after 13N-ammonia PET as well as those with known coronary artery disease (CAD) and all patients who exhibited any coronary artery stenosis ≥ 50% on CCTA and/or regional perfusion abnormalities on 13N-ammonia PET (i.e. ischemia and/or scar as assessed from retention images). Finally, we excluded all patients with an established oral beta-blocker medication. The local ethics committee approved the study (BASEC-Nr. 2023-01220) and granted an exemption for the need for written informed consent.

### CCTA and PET acquisition, reconstruction, and analysis

All patients first underwent CCTA on a 256-slice CT scanner (Revolution CT, GE Healthcare, Waukesha, WI, USA). Examinations included an unenhanced electrocardiogram (ECG)-triggered acquisition for coronary artery calcium (CAC) scoring (i.e., Agatston scoring), followed by the prospectively ECG-triggered contrast-enhanced CCTA. Scan parameters for the unenhanced acquisition included a slice thickness of 2.5 mm, tube voltage of 120 kVp, and tube current of 200 mAs. CCTA was acquired at 75% of the R-R interval, as previously described [7]. Patients with a baseline heart rate (HR) ≥ 67 beats/min received intravenous metoprolol (Beloc Zok, Astra Zeneca, London, UK) at a dose of up to 30 mg [7]. All patients received a 2.5-mg sublingual dose of isosorbide dinitrate (Isoket, Schwarz Pharma, Monheim, Germany). Patients received iodixanol (Visipaque 320; 320 mg I/mL, GE HealthCare) by injection into an antecubital vein followed by a 50 mL injection of saline solution. The contrast agent volume and flow rate were adapted to patients’ body mass index (BMI). A single-beat wide-cone axial cardiac acquisition was performed with the following parameters: collimation, 256 ∙ 0.625 mm; z-axis coverage, 12–16 cm; gantry rotation time, 280 ms. Tube current and voltage were adapted to BMI. Images were reconstructed with a high-definition kernel with a slice thickness of 0.625 mm and increment of 0.625 mm according to clinical routine. A deep learning image reconstruction algorithm (DLIR TrueFidelity, GE HealthCare) was used. CCTA datasets were interpreted at a dedicated workstation (Advantage Workstation 4.7, GE Healthcare). Readers evaluated coronary artery luminal narrowing as a percentage of the vessel diameter by visual estimations for all segments. CAC scoring was performed with commercially available semiautomatic software (SmartScore 4.0, GE HealthCare).

All patients underwent clinically indicated PET MPI using 13N-ammonia within 3 h (1.5 ± 0.6 h) after CCTA. PET was acquired at rest and during pharmacological stress (adenosine infused at 0.14 mg ∙ kg^−1^ ∙ min^−1^ over 6 min or a single bolus injection of 400 µg of regadenoson). All image data were acquired in list mode on a PET/CT (Discovery MI, GE Healthcare) with a BMI-adapted dose of 13N-ammonia (i.e., 165–808 Megabecquerels), as previously reported [8, 9]. The datasets were reconstructed using ordered subset expectation maximization (OSEM, VUE Point HD, 2 iterations, 16 subsets), and a 5 mm Hanning filter and standard decay, scatter and sensitivity corrections (voxel size 2.34, 2.34, 2.80–3.27) were applied. Unenhanced CT was used for attenuation correction of PET/CT datasets. PET image acquisition was acquired in list mode over 14 min. For both stress and rest, dynamic datasets were reconstructed from the first 7 min of acquisition and consisted of 9 frames of 10 s duration, 6 frames of 15 s, 3 frames of 20 s, 2 frames of 30 s, and 1 frame of 120 s. Static and ECG-gated datasets were reconstructed from the last 10 min of the acquisition. MBF during stress (sMBF), at rest (rMBF), myocardial flow reserve (MFR), and the left ventricular ejection fraction and end-diastolic and end-systolic volumes at rest (LVEF_PET baseline,_ LVEDV_PET baseline_, LVESV_PET baseline_) and during vasodilation (LVEF_vasodilation,_ LVEDV_vasodilation_, LVESV_vasodilation_) were calculated using commercially available software (QPET 2017.7 Cedars-Sinai Medical Center, Los Angeles, CA, USA). rMBF and MFR are provided with and without correction for the rate pressure product (RPP).

### Hemodynamic measurements and calculations

HRs were recorded at baseline (HR_CCTA baseline_, i.e. prior to the CCTA acquisition and any beta-blocker administration), during the CCTA acquisition (HR_CCTA_), and prior to the resting (HR_PET baseline_) PET MPI acquisitions. Additionally, the peak HR (HR_vasodilation_) during stress PET MPI was recorded. ΔHR_vasodilation_ was calculated as HR_vasodilation_ minus HR_PET baseline_, reflecting the HR response to vasodilation. To account for inter-individual variability in the response of HR to varying beta-blocker doses, we calculated the HR response to beta-blockers (ΔHR_betablocker_) by subtracting HR_CCTA_ from HR_baseline_, divided by the beta-blocker dose (in mg) per body weight (in kg). The RPP prior to and during vasodilation (RPP_PET baseline_ and RPP_vasodilation_, respectively) was calculated by multiplying the systolic BP with HR_PET baseline_ and HR_vasodilation_, respectively, and ΔRPP_vasodilation_ was calculated as RPP_vasodilation_ minus RPP_PET baseline_. Finally, an index of coronary vascular resistance (CVR) was determined as the ratio of mean arterial blood pressure (MAP, calculated as [diastolic BP + (0.3 ∙ (systolic BP – diastolic BP]) [10] to rMBF (CVR_PET baseline_) and sMBF (CVR_vasodilation_) and ΔCVR_vasodilation_ was calculated as CVR_vasodilation_ minus CVR_PET baseline_.

### Statistical analysis

Statistical analysis was performed using SPSS (version 29.0, IBM Corporation, Armonk, NY, USA). Normally distributed continuous variables are expressed as mean ± standard deviation. Otherwise, the median and interquartile range (IQR; 25th to 75th percentile) are given. Categorical variables are represented as absolute numbers and percentages. Unpaired T-tests were used to compare normally distributed continuous variables. The chi-square test was used to compare dichotomous variables. The Mann–Whitney U test was applied for non-normally distributed variables, and the Jonckheere-Terpstra test for ordered alternatives where the independent variable was of an ordinal type. Spearman correlation analysis was applied for non-normally distributed variables.

Due to this study’s retrospective and observational nature, a random assignment was not possible. Therefore, we performed a propensity-score analysis and 1:1 nearest-neighbour matching based on the baseline variables, which significantly differed between the two groups (i.e. BMI, LVEF, CAC score and the risk factors diabetes and hypertension), using a specified caliper distance of 0.1.

## Results

### Study population

**Fig. 1:**
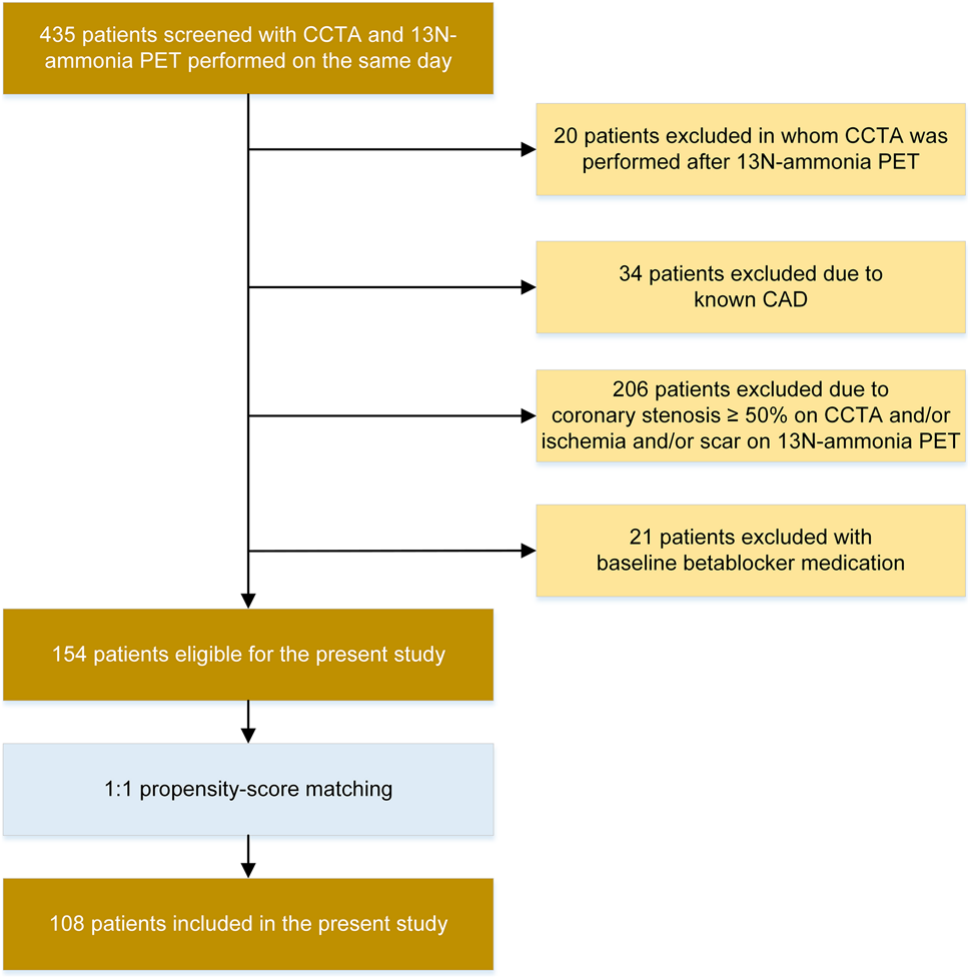
Consolidated standards of reporting trials diagram of patient enrollment

We identified a total of 435 patients who underwent CCTA and 13N-ammonia PET MPI on the same day. A total of 281 (64.6%) of these patients had to be excluded. Details are provided in Fig. 1. One-hundred-and-fifty-four patients were eligible for the present study. Some baseline characteristics differed among those patients with versus without prior application of intravenous beta-blockers, as shown in Supplemental Table S1. The subsequent propensity-score analysis and matching resulted in a cohort of 108 patients, which was used for the final analysis. None of these patients had known cardiomyopathies or valvular disease. Baseline characteristics of these patients are provided in Table 1. Of note, there were no statistically significant differences regarding baseline characteristics between the two groups in this final cohort.

**Table 1:** Baseline characteristics in the overall population and stratified by beta-blocker application

Intravenous application of beta-blockers				
	All patients (n = 108)	No (n = 54)	Yes (n = 54)	p-value
Age (years)	60 ± 11	59 ± 12	60 ± 11	0.537
Female sex	44 (40.7)	22 (40.7)	22 (40.7)	1.000
Body mass index (kg/m^2^)	28.6 ± 6.8	28.2 ± 6.5	28.9 ± 7.2	0.561
Hemoglobin (g/l) ^†^	141 [134–153]	142 [135–155]	140 [130–150]	0.393
Creatinine (µmol/l)	72 [63–84]	72 [63–87]	72 [63–83]	0.536
Glomerular filtration rate (ml/min/1.73m^2^)	103 [81–124]	104 [85–122]	103 [80–129]	0.889
CKD Stage I	73 (67.6)	37 (68.5)	36 (66.7)	0.790
CKD Stage II	32 (29.6)	15 (27.8)	17 (31.5)	
CKD Stage III A	3 (2.8)	2 (3.7)	1 (1.9)	
Cardiovascular risk factors				
Hypertension	44 (40.7)	20 (37.0)	24 (44.4)	0.557
Dyslipidemia	44 (40.7)	18 (33.3)	26 (48.1)	0.170
Diabetes	8 (7.4)	3 (5.6)	5 (9.3)	0.716
Positive family history for CAD	22 (20.4)	11 (20.4)	11 (20.4)	1.000
Smoking	23 (21.3)	9 (16.7)	14 (25.9)	0.347
Cardiac medication				
Antithrombotics	22 (20.4)	15 (27.8)	7 (13.0)	0.093
ACEI/ARB	25 (23.1)	9 (16.7)	16 (29.4)	0.170
Lipid-lowering drugs	20 (18.5)	12 (22.2)	8 (14.8)	0.458
Imaging findings				
CAC score	13 [0–117]	6 [0–84]	25 [0–123]	0.113

Values given are mean ± SD, absolute numbers and percentages in parentheses or median and IQR in brackets

*CKD* chronic kidney disease, *CAD* coronary artery disease, *ACEI* angiotensin converting enzyme inhibitor, *ARB* angiotensin receptor blocker, *CAC* coronary artery calcium

^†^ available in 27 (25%) patients (15 in the group with and 12 in the group without beta-blockers)

### Intravenous beta-blocker application and impact on MBF and heart rates

**Fig. 2:**
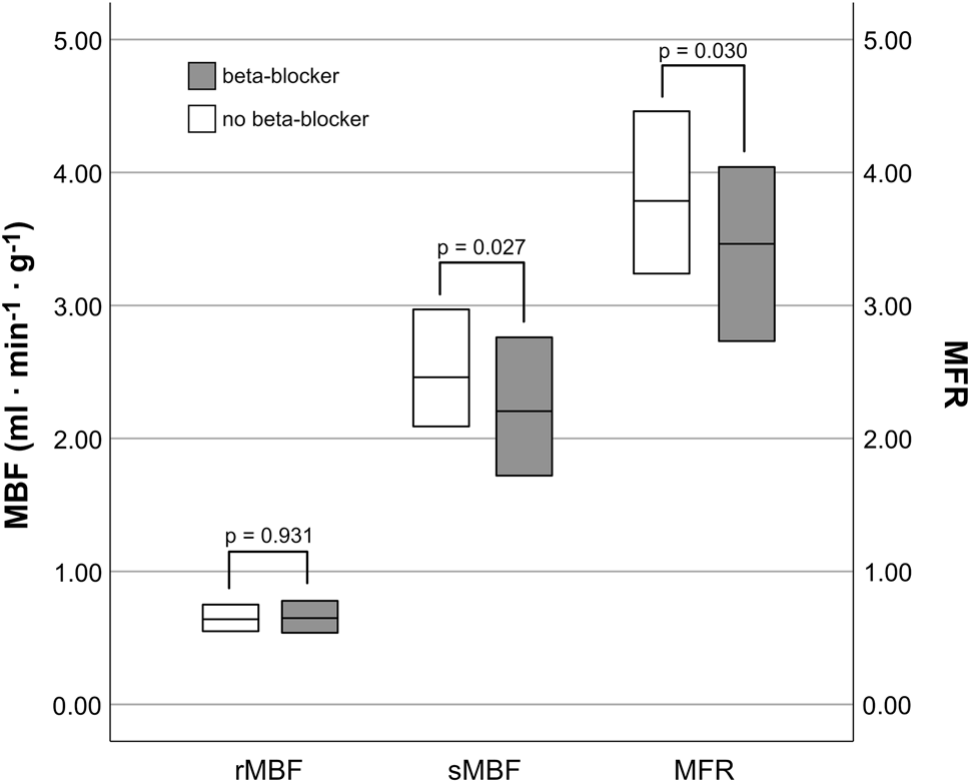
Boxplots of rMBF, sMBF and MFR values of patients in the groups who did versus those who did not receive intravenous beta-blocker. Boxes comprise the IQR and horizontal bars depict the median

MBF measurements and MFR calculations for the overall final cohort and stratified based on whether intravenous beta-blockers were applied or not are provided in Table 2 and Fig. 2. While rMBF did not differ between groups, sMBF and—consequently—MFR values were significantly lower in patients who received beta-blockers versus those who did not. Table 3 provides detailed information on the patients’ hemodynamic parameters recorded before and during the hybrid CCTA/PET examination, as well as the HR response to beta-blockers and vasodilators. Of note, and indicating a lasting beta-blocker effect, patients who had initially received beta-blockers exhibited an attenuated HR- and RPP-response to vasodilators as compared to those who did not receive beta-blockers. Furthermore—and potentially of note—patients who received beta-blockers tended to have smaller LV volumes than patients who did not.

**Table 2:** Myocardial flow parameters in the overall population and stratified by beta-blocker application

Intravenous application of beta-blockers				
	All patients (n = 108)	No (n = 54)	Yes (n = 54)	p-value
rMBF (ml ⋅ min^−1^ ⋅ g^−1^)	0.65 [0.54–0.78]	0.64 [0.55–0.76]	0.65 [0.54–0.78]	0.931
rMBF_uncorrected_ (ml ⋅ min^−1^ ⋅ g^−1^)	0.65 [0.54–0.78]	0.64 [0.55–0.76]	0.65 [0.54–0.78]	0.956
sMBF (ml ⋅ min^−1^ ⋅ g^−1^)	2.36 [1.89–2.94]	2.46 [2.08–2.99]	2.21 [1.72–2.78]	**0.027**
MFR	3.60 [2.99–4.28]	3.79 [3.22–4.46]	3.46 [2.70–4.05]	**0.030**
MFR_uncorrected_	3.60 [2.92–4.29]	3.76 [3.14–4.46]	3.46 [2.70–4.05]	**0.032**

Values given are median and IQR in brackets

*MBF* myocardial blood flow, *MFR* myocardial flow reserve

Statistically significant p-values (i.e. <0.5) are presented in bold

**Table 3:** Hemodynamics in the overall population and stratified by beta-blocker application

Intravenous application of beta-blockers				
	All patients (n = 108)	No (n = 54)	Yes (n = 54)	p-value
CCTA				
HR_CCTA baseline_ (bpm)	70 [60–77]	61 [59–65]	75 [73–80]	** < 0.001**
HR_CCTA_ (bpm)	59 [54–65]	58 [53–63]	61 [54–66]	0.053
ΔHR_CCTA_ (bpm)	-9 [-16 to -4]	-4 [-7 to -1]	-15 [-21 to -11]	** < 0.001**
PET MPI				
LVEF_PET baseline_ (%)	65 ± 7	66 ± 6	65 ± 8	0.700
LVEDV_PET baseline_ (ml)	107 [83–119]	113 [88–123]	102 [78–115]	**0.013**
LVESV_PET baseline_ (ml)	37 [25–46]	39 [27–47]	37 [23–46]	0.220
HR_PET baseline_ (bpm)	61 [55–67]	56 [51–65]	64 [59–68]	** < 0.001**
MAP_PET baseline_ (mmHg)	86 [80–94]	86 [80–98]	86 [89–93]	0.429
RPP_PET baseline_ (bpm ∙ mmHg)	7188 [6370–8645]	6795 [5770–8240]	7310 [6525–8674]	0.128
CVR_PET baseline_ (mmHg ∙ ml^−1^ ∙ min^−1^ ∙ g^−1^)	135 [113–158]	138 [116–155]	129 [111–159]	0.570
LVEF_vasodilation_ (%)	66 ± 8	66 ± 8	67 ± 8	0.815
LVEDV_vasodilation_ (ml)	116 [92–131]	123 [97–140]	110 [86–122]	**0.009**
LVESV_vasodilation_ (ml)	38 [27–50]	43 [28–53]	36 [26–47]	0.088
HR_vasodilation_ (bpm)	84 [75–93]	84 [73–95]	84 [76–90]	0.791
MAP_vasodilation_ (mmHg)	84 [77–95]	87 [79–97]	82 [75–90]	0.134
RPP_vasodilation_ (bpm ∙ mmHg)	9516 [8418–11539]	10,080 [8354–11700]	9234 [8450–11551]	0.450
CVR_vasodilation_ (mmHg ∙ ml^−1^ ∙ min^−1^ ∙ g^−1^)	35 [28–47]	35 [28–43]	37 [29–52]	0.144
ΔLVEF_vasodilation_ (%)	1 ± 5	1 ± 4	2 ± 5	0.334
ΔLVEDV_vasodilation_ (ml)	12 [6–16]	12 [6–18]	10 [7–16]	0.538
ΔLVESV_vasodilation_ (ml)	2 [− 2 to 6]	2 [− 1 to 7]	2 [− 3 to 5]	0.148
ΔHR_vasodilation_ (bpm)	23 [14–30]	27 [16–36]	21 [13–28]	**0.036**
ΔMAP_vasodilation_ (mmHg)	− 1 [-7 to 5]	1 [− 6 to 5]	− 2 [− 7 to 3]	0.327
ΔRPP_vasodilation_ (bpm ∙ mmHg)	2289 [1426–4209]	3119 [1640–4567]	2057 [1103–3643]	**0.040**
ΔCVR_vasodilation_ (mmHg ∙ ml^−1^ ∙ min^−1^ ∙ g^−1^)	− 97 [− 115 to − 76]	− 100 [− 118 to − 78]	− 95 [− 112 to − 76]	0.257

Values given are median and IQR in brackets

*CCTA* coronary CT angiography, *HR* heart rate, *bpm* beats per minute, *LVEF* left ventricular ejection fraction, *LVEDV* left ventricular end-diastolic volume, *LVESV* left ventricular end-systolic volume, *MAP* mean arterial pressure, *RPP* rate pressure product, *CVR* coronary vascular resistance

﻿Statistically significant p-values (i.e. <0.5) are presented in bold

### Dose dependencies

**Fig. 3:**
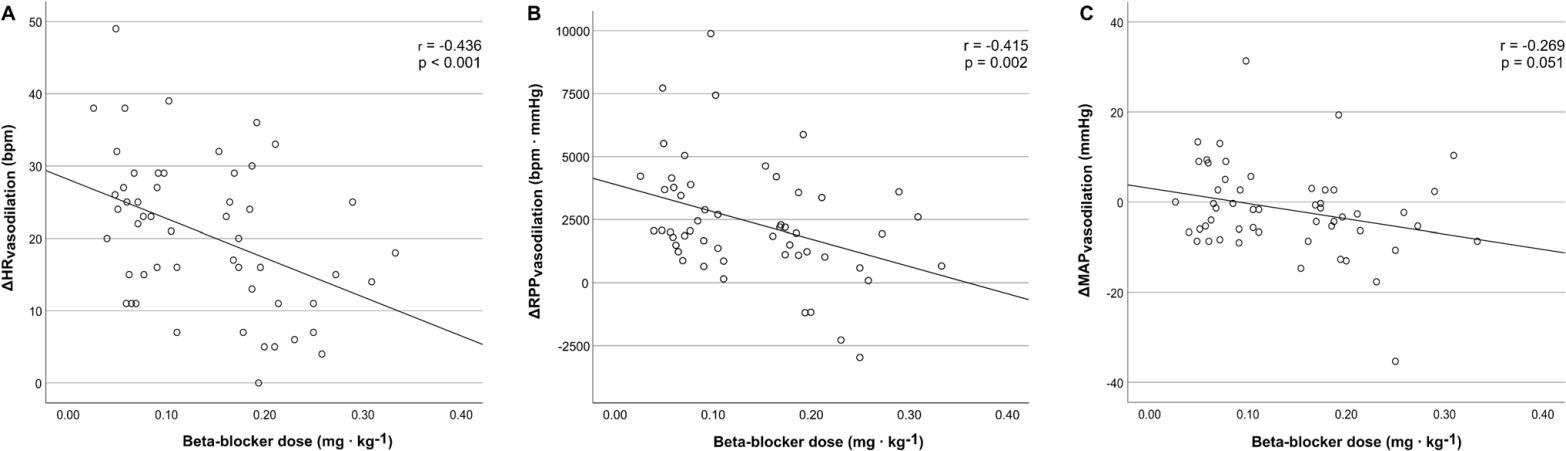
Scatterplots for ΔHR_vasodilation_ (**A**), ΔRPP_vasodilation_ (**B**), and ΔMAP_vasotilation_ (**C**), in relation to the applied beta-blocker dose

Among patients in whom beta-blocker was applied, the median dose was 0.11 mg∙kg^−1^ (IQR 0.07 to 0.20). There was a weak but statistically significant and comparable correlation between beta-blocker dose per body weight and ΔHR_vasodilation_ (r = − 0.436, p < 0.001, Fig. 3A) and ΔRPP_vasodilation_ (r = − 0.415, p = 0.002, Fig. 3B), and a trend for the correlation between beta-blocker dose per body weight and ΔMAP_vasodilation_ (r = − 0.269, p = 0.051, Fig. 3C). However, we did not find any correlations between beta-blocker dose per body weight and rMBF (p = 0.392), sMBF (p = 0.198), or MFR (p = 0.538).

**Fig. 4:**
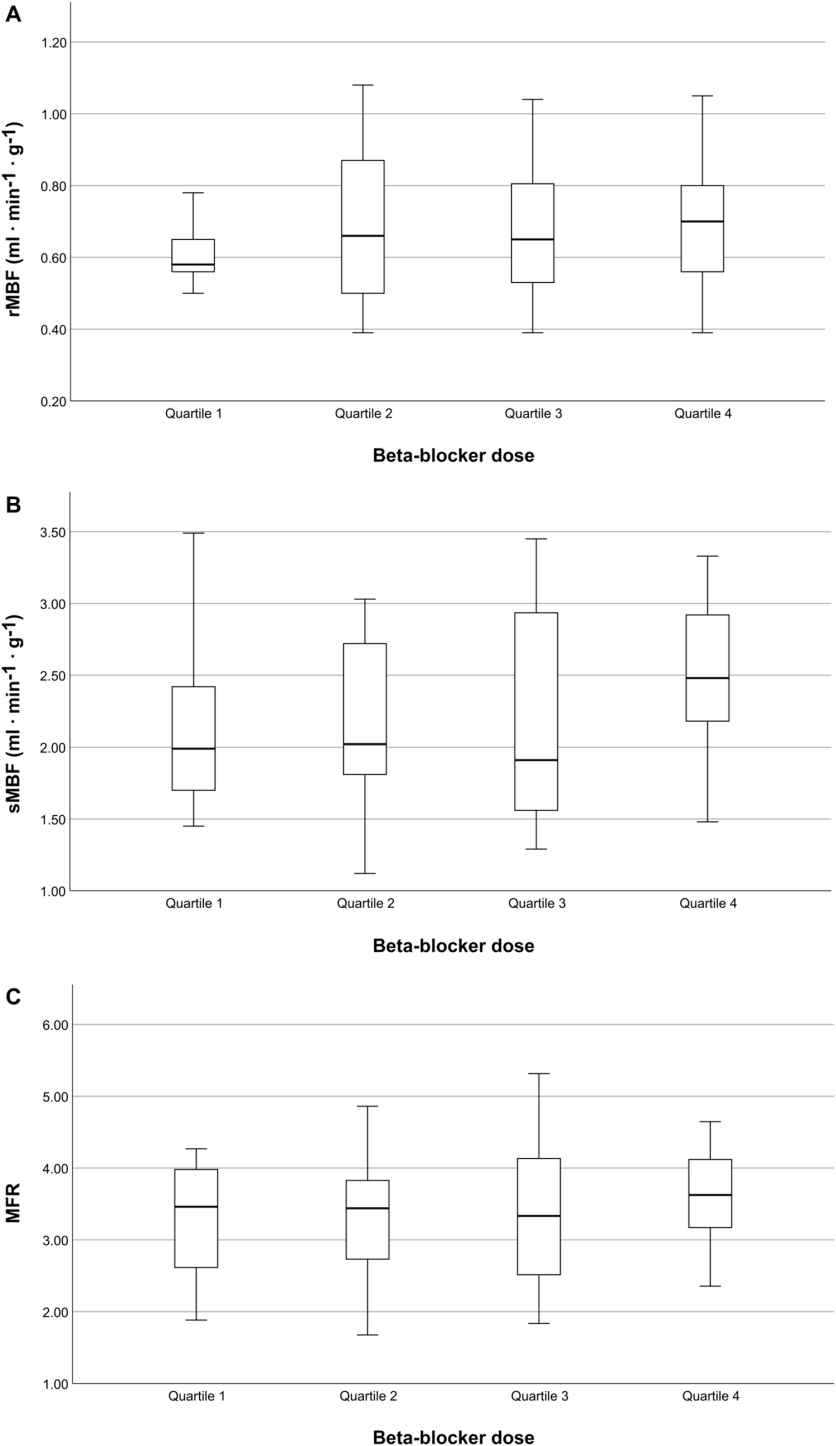
Boxplots of rMBF (**A**), sMBF (**B**) and MFR (**C**) values of patients grouped into quartiles based on the received beta-blocker dose. Boxes comprise the IQR and horizontal bars depict the median

Similarly, when grouping patients into quartiles based on the received beta-blocker dose per weight, there were no statistically significant differences among groups regarding rMBF (p = 0.408), sMBF (p = 0.148), or MFR (p = 0.492) (Fig. 4, A-C).

### Impact on patient classification

Previous studies have proposed a threshold for sMBF and MFR of 1.8 ml min^−1^∙g^−1^ and 2.0, respectively, for the diagnosis of coronary microvascular dysfunction (CMD) [11, 12]. When applying these thresholds to the patients in the present study, we found that, if stratified by sMBF, 16 (29.6%) patients were below the threshold in the group who received beta-blockers and 6 (11.1%) patients in the group who did not (p = 0.017). When stratified by MFR, 3 (5.6%) patients were below the threshold in the group who received beta-blockers, whereas no patients had an MFR < 2.0 in the group who did not receive beta-blockers (p = 0.079).

## Discussion

In this study, we utilized quantitative 13N-ammonia PET MPI with vasodilator stress to explore the effects of acute intravenous beta-blocker administration on quantitative MBF. Our results demonstrate that beta-blockers significantly influence sMBF and MFR, with reductions of 10.2% and 8.7%, respectively, in patients who received beta-blockers compared to those who did not. Interestingly, rMBF remained consistent across both groups. These findings indicate that although the coronary circulation at rest is unaffected by beta-blockers, its ability to react to vasodilators is reduced. Additionally, while there was a weak, but notable inverse correlation between the dosage of beta-blockers and the HR and RPP responses to vasodilators, our findings do not suggest a direct dose dependency between beta-blockers and the changes observed in sMBF or MFR. The latter indicates that the impact of beta-blockers on stress responses may be more complex and not solely dependent on the dosage administered.

Although no studies have yet explored the effects of acute, i.e. same-day intravenous administration of beta-blockers on quantitative MBF as measured by PET MPI, several historical studies have approached this subject from various perspectives, employing different methodologies and yielding mixed, and at times, contradictory results:

In 1997, Böttcher et al. conducted a study with ten healthy volunteers using 13N-ammonia PET and dipyridamole-induced vasodilation, revealing that beta-blockade influenced both rMBF and sMBF [13]. Their findings showed a 16% decrease in rMBF following oral administration of metoprolol, accompanied by a 26% increase in sMBF. This resulted in a surprising 46% increase in MFR, leading the authors to conclude that beta-blockers do not blunt the pharmacological effects of dipyridamole. In contrast, our study presents markedly different outcomes, potentially attributable to methodological variations and differences in patient demographics. Böttcher et al. employed dipyridamole as a vasodilator and used oral beta-blockers in a small cohort of young, healthy volunteers (mean age 24 ± 5 years), rendering a direct comparison between this and our study difficult if not impossible.

By contrast, in their study with 25 patients diagnosed with CAD undergoing SPECT imaging with dipyridamole, Taillefer et al. discovered that the administration of intravenous beta-blockers compromised the sensitivity of MPI for detecting perfusion defects [14]. They noted a significant reduction in both the extent and severity of perfusion defects, indicating that acute beta-blocker use could obscure the detection and assessment of CAD during SPECT MPI. Consequently, the authors recommended discontinuing beta-blockers prior to testing to prevent diagnostic inaccuracies. Our findings align with, and potentially validate, those of Taillefer et al., suggesting that the decreased sensitivity observed in SPECT could be attributed to a reduction in MFR. This reduction likely minimizes the detectable differences in myocardial perfusion, thus impacting the diagnostic sensitivity of SPECT.

Finally, our results are largely consistent with a more recent study by Hoffmeister et al., which examined MBF in 20 patients using N13-ammonia PET both before and after the withdrawal of baseline oral beta-blocker therapy [15]. In that study, discontinuing beta-blockers led to a significant increase in sMBF during adenosine-induced vasodilation in patients with CAD. Although the study focused on the effects of withdrawal of baseline beta-blocker medication, the observed increase in sMBF (i.e. an increase of 7.2% after withdrawal) closely mirrors the outcomes of our current research, albeit slightly less pronounced.

When comparing our results to previous studies, it is important to recognize that, contrary to the vast majority of previous studies, the findings from the current study result from a relatively healthy population. Thus, the effects of concomitant cardiac pathologies, including obstructive and/or hemodynamically significant CAD, are eliminated, providing clearer and less confounded insights into how beta-blockers affect coronary physiology. Interestingly, we found no correlation between the dose of beta-blockers and MBF or MFR nor any dose thresholds at which beta-blockers start to have an effect. On the other hand, we found a dose dependency between beta-blockers and the hemodynamic response to vasodilation. This reflects the complex interplay between beta-blockers and MBF, which may be attributable to several pharmacological and physiological, potentially even non-uniform, mechanisms: Firstly, a beta-blocker-induced reduction in HR and myocardial contractility during vasodilation-induced stress attenuates the myocardial MBF demand, resulting in lower sMBF and MFR. This is in line with our finding of diminished HR response to vasodilation. A blunted beta-adrenergic response to stress may additionally confer to this finding. Secondly, it has been proposed that beta-blockers lead to an increase in CVR during hyperemia due to unopposed alpha-adrenergic vasomotor tone [16]. The results from our study, however, do not lend support to this hypothesis as we found that CVR was comparable between both groups both before und during pharmacological vasodilation. Furthermore, it may be hypothesized that there is a direct, and likely dose-independent, interaction between beta-blockers and vasodilators as beta-2 receptors are coupled to the same types of stimulatory G-proteins as the A2 receptors for adenosine and regadenoson. Therefore, it is possible that the cAMP response from A2 receptor stimulation is attenuated after beta-blockade. If this is the mechanism, it suggests that a similar interaction may occur between adenosine or regadenoson and beta-blockade. Finally, it must be taken into consideration that the hyperemic MBF response after beta-blocker is subject to inter-individual variation. For example, in the study by Hoffmeister et al., 15% of patients did not exhibit an increase, but a decrease in MBF. This observation underlines the complexity of the aforementioned interaction between beta-blockers and vasodilator MBF. Based on our results, it could also be hypothesized that a patient’s individual sympathetic activity may be predictive of the impact of beta-blockers on MBF. In our study, those patients who needed beta-blockers to reach the target HR for CCTA also exhibited again higher HRs prior to PET (HR_PET baseline_). This may be reflective of an overall elevated sympathetic tone. Although speculative at this point, the fact that patients who received beta-blockers tended to have smaller LV volumes (and, thus, smaller stroke volumes), may be lending support to this thesis. By blocking beta-adrenergic receptors, these medications could have minimized the cardiovascular response during stress in this specific patient group. Conversely, the lack of change in rMBF suggests that beta-blockers have minor impact on basal coronary artery function or flow, thus leaving rMBF generally unaffected.

Although we can only hypothesize about the precise mechanisms, the findings from our study underscore the intricate interplay between beta-blockers and vasodilators in influencing coronary hemodynamics during MPI. Importantly, our findings shed light on the extent of these effects through quantification of the MBF, allowing us to draw conclusions about potential clinical implications. At first, the latter may appear modest, as evidenced by the approximately 10% reduction in stress MBF (sMBF) and myocardial flow reserve (MFR), especially when considered alongside the inherent day-to-day biological and methodological variability in MBF measurements, which can fluctuate by up to ± 20% [17]. Nonetheless, for facilities that conduct same-day hybrid imaging with CCTA and PET, it is crucial to consider how protocol-driven beta-blocker administration might influence sMBF and MFR. This consideration is particularly pertinent for interpreting results in patients whose MBF measurements are near clinical decision thresholds, when, for example, evaluating suspected CMD in the absence of obstructive CAD and myocardial diseases or for risk stratification purposes.

## Conclusion

Acute intravenous beta-blocker administration impacts MBF, leading to a slight reduction in sMBF and MFR in patients without obstructive CAD. In contrast, rMBF appears unaffected, suggesting that beta-blockers primarily affect the coronary capacity to respond to vasodilators.

### Limitations and strengths

This is a retrospective study with all the inherent limitations of such a study design. Although, we aimed to mitigate this potential limitation by applying a propensity score matching, yielding two highly comparable patient groups for comparison and rendering bias from baseline characteristics unlikely, we cannot with certainty exclude a possible selection bias and residual confounding. Additionally, the study’s single-center nature may limit generalizability and, of note, the results and conclusions drawn are not directly applicable to patients who chronically take oral beta-blockers and in whom the decision whether or not to interrupt the medication prior to PET MPI should be made. Future multicenter studies would help validate the results and should ideally assess the impact of acute beta-blocker application on diagnostic accuracy of PET MPI through a cross-over study design. Furthermore, we excluded all patients with known CAD, with potentially flow-limiting coronary stenoses and those with myocardial perfusion defects. While this constitutes a considerable strength of this research by eliminating potential confounding co-disease enabling unbiased insights into the physiological interplay between beta-blockers, hemodynamics and coronary flow, it also limits our ability to extrapolate our findings to patients with disease.

## Supplementary Information

Below is the link to the electronic supplementary material.

Supplementary file1 (DOCX 20 KB)

Supplementary file2 (DOCX 20 KB)

## Data Availability

No datasets were generated or analysed during the current study.

## References

[1] Kajander S, Joutsiniemi E, Saraste M, Pietila M, Ukkonen H, Saraste A, Sipila HT, Teras M, Maki M, Airaksinen J, Hartiala J, Knuuti J (2010) Cardiac positron emission tomography/computed tomography imaging accurately detects anatomically and functionally significant coronary artery disease. Circulation 122(6):603–613. 10.1161/CIRCULATIONAHA.109.915009 20660808 10.1161/CIRCULATIONAHA.109.915009

[2] Knuuti J, Wijns W, Saraste A, Capodanno D, Barbato E, Funck-Brentano C, Prescott E, Storey RF, Deaton C, Cuisset T, Agewall S, Dickstein K, Edvardsen T, Escaned J, Gersh BJ, Svitil P, Gilard M, Hasdai D, Hatala R, Mahfoud F, Masip J, Muneretto C, Valgimigli M, Achenbach S, Bax JJ, E.S.D. Group (2019) 2019 ESC guidelines for the diagnosis and management of chronic coronary syndromes: the task force for the diagnosis and management of chronic coronary syndromes of the European society of cardiology (ESC). Eur Heart J 41(3):407–477. 10.1093/eurheartj/ehz425 10.1093/eurheartj/ehz425 31504439

[3] Pazhenkottil AP, Benz DC, Grani C, Madsen MA, Mikulicic F, von Felten E, Fuchs TA, Moch BH, Stehli J, Luscher TF, Gaemperli O, Buechel RR, Kaufmann PA (2018) Hybrid SPECT perfusion imaging and coronary CT angiography: long-term prognostic value for cardiovascular outcomes. Radiology 288(3):694–702. 10.1148/radiol.2018171303 29969066 10.1148/radiol.2018171303

[4] Narula J, Chandrashekhar Y, Ahmadi A, Abbara S, Berman DS, Blankstein R, Leipsic J, Newby D, Nicol ED, Nieman K, Shaw L, Villines TC, Williams M, Hecht HS (2021) SCCT 2021 expert consensus document on coronary computed tomographic angiography: a report of the society of cardiovascular computed tomography. J Cardiovasc Comput Tomogr 15(3):192–217. 10.1016/j.jcct.2020.11.001 33303384 10.1016/j.jcct.2020.11.001 PMC8713482

[5] Buechel RR, Husmann L, Herzog BA, Pazhenkottil AP, Nkoulou R, Ghadri JR, Treyer V, von Schulthess P, Kaufmann PA (2011) Low-dose computed tomography coronary angiography with prospective electrocardiogram triggering: feasibility in a large population. J Am Coll Cardiol 57(3):332–336. 10.1016/j.jacc.2010.08.634 21232672 10.1016/j.jacc.2010.08.634

[6] Zoghbi GJ, Dorfman TA, Iskandrian AE (2008) The effects of medications on myocardial perfusion. J Am Coll Cardiol 52(6):401–416. 10.1016/j.jacc.2008.04.035 18672159 10.1016/j.jacc.2008.04.035

[7] Benz DC, Grani C, Hirt Moch B, Mikulicic F, Vontobel J, Fuchs TA, Stehli J, Clerc OF, Possner M, Pazhenkottil AP, Gaemperli O, Buechel RR, Kaufmann PA (2016) Minimized radiation and contrast agent exposure for coronary computed tomography angiography: first clinical experience on a latest generation 256-slice scanner. Acad Radiol 23(8):1008–1014. 10.1016/j.acra.2016.03.015 27174030 10.1016/j.acra.2016.03.015

[8] Benz DC, Kaufmann PA, von Felten E, Benetos G, Rampidis G, Messerli M, Giannopoulos AA, Fuchs TA, Grani C, Gebhard C, Pazhenkottil AP, Flammer AJ, Kaufmann PA, Buechel RR (2021) Prognostic value of quantitative metrics from positron emission tomography in ischemic heart failure. JACC Cardiovasc Imaging 14(2):454–464. 10.1016/j.jcmg.2020.05.033 32771569 10.1016/j.jcmg.2020.05.033

[9] Degtiarova G, Garefa C, Boehm R, Ciancone D, Sepulcri D, Gebhard C, Giannopoulos AA, Pazhenkottil AP, Kaufmann PA, Buechel RR (2023) Radiomics for the detection of diffusely impaired myocardial perfusion: a proof-of-concept study using 13N-ammonia positron emission tomography. J Nucl Cardiol. 10.1007/s12350-022-03179-y 36600174 10.1007/s12350-022-03179-y PMC10371953

[10] Vincenti G, Quercioli A, Zaïdi H, Nkoulou R, Dewarrat S, Rager O, Ambrosio G, Seimbille Y, Mach F, Ratib O, Schindler TH (2010) Combined evaluation of myocardial perfusion and coronary morphology in the identification of subclinical CAD. Radiation exposure of 13N-ammonia PET/CT. Nuklearmedizin 49(5):173–182. 10.3413/nukmed-0312 20664888 10.3413/nukmed-0312

[11] Schindler TH, Dilsizian V (2020) Coronary microvascular dysfunction: clinical considerations and noninvasive diagnosis. JACC Cardiovasc Imaging 13(1 Pt 1):140–155. 10.1016/j.jcmg.2018.11.036 30982670 10.1016/j.jcmg.2018.11.036

[12] Schindler TH, Fearon WF, Pelletier-Galarneau M, Ambrosio G, Sechtem U, Ruddy TD, Patel KK, Bhatt DL, Bateman TM, Gewirtz H, Shirani J, Knuuti J, Gropler RJ, Chareonthaitawee P, Slart R, Windecker S, Kaufmann PA, Abraham MR, Taqueti VR, Ford TJ, Camici PG, Schelbert HR, Dilsizian V (2023) Myocardial perfusion PET for the detection and reporting of coronary microvascular dysfunction: a JACC: cardiovascular imaging expert panel statement. JACC Cardiovasc Imaging 16(4):536–548. 10.1016/j.jcmg.2022.12.015 36881418 10.1016/j.jcmg.2022.12.015

[13] Böttcher M, Czernin J, Sun K, Phelps ME, Schelbert HR (1997) Effect of beta 1 adrenergic receptor blockade on myocardial blood flow and vasodilatory capacity. J Nucl Med 38(3):442–446 9074535

[14] Taillefer R, Ahlberg AW, Masood Y, White CM, Lamargese I, Mather JF, McGill CC, Heller GV (2003) Acute beta-blockade reduces the extent and severity of myocardial perfusion defects with dipyridamole Tc-99m sestamibi SPECT imaging. J Am Coll Cardiol 42(8):1475–1483. 10.1016/s0735-1097(03)01046-5 14563595 10.1016/s0735-1097(03)01046-5

[15] Hoffmeister C, Preuss R, Weise R, Burchert W, Lindner O (2014) The effect of beta blocker withdrawal on adenosine myocardial perfusion imaging. J Nucl Cardiol 21(6):1223–1229. 10.1007/s12350-014-9952-y 25124825 10.1007/s12350-014-9952-y PMC4228113

[16] Kern MJ, Ganz P, Horowitz JD, Gaspar J, Barry WH, Lorell BH, Grossman W, Mudge GH Jr (1983) Potentiation of coronary vasoconstriction by beta-adrenergic blockade in patients with coronary artery disease. Circulation 67(6):1178–1185. 10.1161/01.cir.67.6.1178 6133636 10.1161/01.cir.67.6.1178

[17] Kitkungvan D, Johnson NP, Roby AE, Patel MB, Kirkeeide R, Gould KL (2017) Routine clinical quantitative rest stress myocardial perfusion for managing coronary artery disease: clinical relevance of test-retest variability. JACC Cardiovasc Imaging 10(5):565–577. 10.1016/j.jcmg.2016.09.019 28017383 10.1016/j.jcmg.2016.09.019

