# Supplementary material for: Effect of acute intravenous beta-blocker administration on myocardial blood flow during same-day hybrid CCTA/PET imaging
Source: Int J Cardiovasc Imaging. 2024 Aug 5;40(10):2203–12. doi: 10.1007/s10554-024-03212-w (PMC11499384; doi:10.1007/s10554-024-03212-w)
Supplement: Supplementary file 1 — Supplementary file1 (DOCX 20 KB) [file 10554_2024_3212_MOESM1_ESM.docx]

**TABLES**

**Table S1. Baseline characteristics in the overall population and stratified by beta-blocker application**

|  |  | Intravenous application of betablockers | |  |
| --- | --- | --- | --- | --- |
|  | All patients (n=154) | No (n=75) | Yes (n=79) | p-value |
| Age (years) | 59 ± 11 | 58 ± 12 | 60 ± 11 | 0.209 |
| Female sex | 58 (37.7) | 25 (33.3) | 29 (36.7) | 0.320 |
| Body mass index (kg/m^2^) | 28.6 ± 7.3 | 27.1 ± 6.5 | 30.0 ± 7.8 | **0.012** |
| Creatinine clearance (ml/min/1.73m^2^) | 103 [82-127] | 100 [80-122] | 104 [88-137] | 0.254 |
| Cardiovascular risk factors |  |  |  |  |
| Hypertension | 64 (41.6) | 23 (30.7) | 41 (51.9) | **0.009** |
| Dyslipidemia | 55 (35.7) | 22 (29.3) | 33 (41.8) | 0.131 |
| Diabetes | 26 (16.9) | 4 (5.3) | 22 (27.8) | **<0.001** |
| Positive family history for CAD | 28 (18.2) | 12 (16.0) | 16 (20.3) | 0.536 |
| Smoking | 25 (16.2) | 10 (13.3) | 15 (19.0) | 0.387 |
| Cardiac medication |  |  |  |  |
| Antithrombotics | 27 (17.5) | 17 (22.7) | 10 (12.7) | 0.138 |
| Beta blockers | 0 (0) | 0 (0.0) | 0 (0.0) |  |
| ACEI/ARB | 28 (18.2) | 10 (13.3) | 18 (22.8) | 0.147 |
| Lipid-lowering drugs | 30 (19.5) | 14 (18.7) | 16 (20.3) | 0.841 |
| Imaging findings |  |  |  |  |
| LVEF (%) | 65 ± 8 | 64 ± 8 | 67 ± 8 | **0.051** |
| CAC score^†^ | 7 [0-94] | 2 [0-26] | 18 [0-119] | **0.030** |
| rMBF (ml ∙ min^-1^ ∙ g^-1^) | 0.64 [0.56-0.75] | 0.63 [0.56-0.75] | 0.65 [0.54-0.76] | 0.964 |
| rMBF_uncorrected_ (ml ∙ min^-1^ ∙ g^-1^) | 0.65 [0.56-0.76] | 0.64 [0.56-0.75] | 0.65 [0.56-0.77] | 0.974 |
| sMBF (ml ∙ min^-1^ ∙ g^-1^) | 2.39 [1.90-2.88] | 2.51 [2.09-3.02] | 2.24 [1.75-2.76] | **0.013** |
| MFR | 3.64 [3.05-4.27] | 3.82 [3.24-4.57] | 3.49 [2.91-3.98] | **0.007** |
| MFR_uncorrected_ | 3.53 [2.98-4.27] | 3.82 [3.09-4.53] | 3.40 [2.85-3.96] | **0.007** |

Values given are mean ± SD, absolute numbers and percentages in parentheses or median and IQR in brackets. CAD = coronary artery disease; ACEI = angiotensin converting enzyme inhibitor; ARB = angiotensin receptor blocker; LVEF = left ventricular ejection fraction; CAC = coronary artery calcium; MBF = myocardial blood flow; MFR = myocardial flow reserve.
